# Relationships between Personality and Cognitive Ability: A Facet-Level Analysis

**DOI:** 10.3390/jintelligence6020028

**Published:** 2018-05-18

**Authors:** Beatrice Rammstedt, Clemens M. Lechner, Daniel Danner

**Affiliations:** 1 GESIS—Leibniz Institute for the Social Sciences, P.O. Box 12 21 55, D-68072 Mannheim, Germany; clemens.lechner@gesis.org; 2 University of Applied Labor Studies, Seckenheimer Landstr. 16, D-68163 Mannheim, Germany; ddanner@gmx.de

**Keywords:** cognitive skills, personality, Big Five, facets, fluid and crystallized intelligence

## Abstract

A growing body of research supports the notion that cognitive abilities and personality are systematically related. However, this research has focused largely on global personality dimensions and single—often equally global—markers of cognitive ability. The present study offers a more fine-grained perspective. Specifically, it is one of the first studies to comprehensively investigate the associations between both fluid and crystallized intelligence with Big Five personality domains as well as their facets. Based on a heterogeneous sample of the adult population in Germany (*N* = 365), our study yielded three key findings. First, personality was more strongly related to crystallized intelligence than to fluid intelligence. This applied both to the total variance explained and to the effect sizes of most of the Big Five domains and facets. Second, facets explained a larger share of variance in both crystallized and fluid intelligence than did domains. Third, the associations of different facets of the same domain with cognitive ability differed, often quite markedly. These differential associations may substantially reduce—or even suppress—the domain-level associations. Our findings clearly attest to the added value of a facet-level perspective on the personality–cognitive ability interface. We discuss how such a fine-grained perspective can further theoretical understanding and enhance prediction.

## 1. Introduction

The relationships of cognitive abilities to “non-ability” traits such as personality have long been of interest to the study of individual differences. Some of the most eminent early theorists in this area shared the view that personality traits deserved to be studied alongside cognitive abilities, and they attempted to integrate both constructs into conceptual frameworks of human abilities. For example, Thorndike [1] suggested that socially valued traits such as virtues and social skills were likely to be positively related to cognitive ability, forming a positive manifold. Wechsler [2] proposed that a range of what he summarized as “non-intellective traits” should be included in IQ tests, because they might enhance the prediction of real-life performance. Similarly, Vernon’s [3] model of the structure of educational abilities included an “X” factor as a placeholder for various personality traits and interests.

Despite these ample theoretical foundations, few systematic empirical efforts to unravel the relations between cognitive ability and personality were made in the years that followed [4,5]. In recent decades, however, a growing body of evidence has accumulated that supports the notion that cognitive abilities and personality are systematically related. A large part of this research has used the Big Five framework as a guidepost. The Big Five is currently the most extensively validated and widely used model of personality, and, as such, it has greatly facilitated the systematic study of personality–ability relations. Results of numerous studies, including meta-analyses (e.g., [6]) have demonstrated that there are robust—albeit only modest—associations between several of the Big Five personality traits and measures of cognitive ability. The proportion of variance in cognitive ability that can be jointly explained by Big Five personality traits typically ranges between five and ten percent [7].

Of the Big Five domains, Openness to Experience and Emotional Stability are the ones that show the strongest and most robust positive links to measures of cognitive ability (see, e.g., meta-analytical results by [5,6,8,9,10]). Two mechanisms are typically invoked to explain these two most robust associations. First, certain personality traits are assumed to facilitate the acquisition of knowledge and abilities [5]. This applies especially to the domain of Openness, which includes characteristics such as intellectual curiosity. According to investment theory [8,11], such characteristics help individuals to translate their basic cognitive abilities into knowledge acquisition. Second, personality traits may be indirectly related to cognitive abilities through their influence on individuals’ behavior and performance in the test-taking situation as such. This is most evident for Emotional Stability, as some research suggests that high-stakes test situations, in particular, might evoke higher test anxiety in neurotic individuals, which in turn might interfere with the cognitive processing of the test tasks, thereby hampering test performance [12].

A correlation of Extraversion with cognitive ability has also occasionally been reported. However, the sign of the correlation coefficient has been inconsistent across studies [6,9,13,14,15,16]. The test situation itself has also been suggested as the mechanism underlying this association. It has been argued that the association might be a function of whether the cognitive ability test is conducted under timed or untimed conditions, as some studies have reported that extraverts performed better on timed tasks [17], whereas introverts tended to score higher on tasks requiring insight and reflection [18].

Several studies have also consistently reported a negative association between Conscientiousness and measures of cognitive ability (e.g., [7,9,19,20,21]). This appears contradictory at first sight, given that both variables are positively linked to work-related outcomes (e.g., [22,23,24]). Some researchers have tried to explain this finding with reference to methodological artifacts caused by sampling bias (e.g., [20,25]), as most of the studies were based on college student populations only (see [7]), and thus on respondents with high cognitive ability and high Conscientiousness (see [26]). From a more substantive perspective, it has been argued that the negative correlation between Conscientiousness and cognitive ability reflects a compensatory process whereby individuals with lower levels of cognitive ability develop higher levels of Conscientiousness in order to make up for their cognitive disadvantages [9,27]^1^.

Besides these linear associations, some studies have suggested that the association between personality and cognitive ability can better be explained by non-linear associations (for a discussion, see [4]). Recent research findings have shown, for example, that the associations of Emotional Stability and Openness with cognitive ability are indeed primarily triggered by comparatively low cognitive skills of persons who are low in Emotional Stability or Openness [30].

### 1.1. Personality and Cognitive Ability: Toward a More Fine-Grained Perspective

The aforementioned links between personality—especially Openness and Emotional Stability—and cognitive ability are now empirically well established. Yet, these links may conceal considerable complexity and diversity that lies behind global associations. This is because both cognitive ability and individual personality traits comprise different (factor-analytically derived) dimensions or facets that can be modeled hierarchically. Of course, different (sub-)dimensions of cognitive ability (e.g., verbal and numerical abilities and the overarching *g* factor) are well known to be positively interrelated (“positive manifold”), as are different facets of a given personality dimension (e.g., the industriousness and self-control facets of Conscientiousness). Nonetheless, different aspects of cognitive ability and facets of personality might be differentially related to each other [5,21].

With regard to cognitive ability, we adopt in this paper the distinction between fluid and crystallized intelligence. Fluid intelligence describes general abilities such as reasoning and problem-solving, whereas crystallized intelligence reflects acquired skills, knowledge and experience. This distinction goes back to Cattell, who built on work by Hebb (see [31] for a discussion). It remains one of the most common distinctions in research on cognitive abilities [32], and it is also reflected in later hierarchical cognitive ability models such as the Cattell-Horn-Carroll (CHC) model. The CHC model describes fluid and crystallized intelligence as broad classes of abilities on the second stratum, subordinate to general cognitive ability (*g*; [33,34]). Fluid and crystallized intelligence develop differently over the lifespan, and they interact. Whereas fluid intelligence develops rapidly early in life and peaks in young adulthood, crystallized intelligence is thought to increase more or less constantly over the life course, depending on how fluid intelligence is invested.

To the extent that fluid and crystallized intelligence reflect specific abilities that have partly different physiological substrates and reflect distinct developmental processes [32], their associations with the Big Five personality domains and facets are likely to differ. For Openness, for example, it has been proposed that open individuals, being more intellectually curious, are more motivated to engage in intellectual activities, which leads them to expand their crystallized intelligence (see [8,35]). As fluid intelligence is less sensitive to intellectual investments and less influenced by learning, it should exhibit comparatively weaker relationships to Openness. Several findings suggesting that Openness is correlated particularly with crystallized intelligence but to a lesser degree with fluid intelligence support this notion [21,36,37,38,39].

Like cognitive ability, personality can be viewed as a multi-faceted construct that can be modeled hierarchically with different levels of aggregation [40,41]. In recent years, researchers have placed increasing emphasis on examining more specific facets of the broad Big Five domains [41,42,43]. This growing body of research suggests that narrow facets of a personality domain not only have greater predictive power than global domains, but they also exhibit *differential* associations with different outcome variables. For these reasons, some researchers (e.g., [42]) even recommend that only the facet level of the Big Five should be investigated, as this allows insights to be gained into causal relationships, thus explaining associations found at the level of personality domains. For example, based on analyses of the short version of the Big Five Inventory–2 (BFI-2-S; [44]), Rammstedt and colleagues [45] showed that—of the three facets of Extraversion—*energy*, in particular, was related to life satisfaction and health, whereas *assertiveness* and *sociability* showed weaker relations to those outcomes. Similarly, Roberts and colleagues [46] demonstrated that the *responsibility* facet of Conscientiousness was more predictive of health-related behavior than any other facet of this domain.

This promising research on differential associations of outcome variables with personality facets begs the question of whether different facets of the same personality domain also show different links to (different types of) cognitive ability. Attending to the possibility of differential relationships among different types of cognitive ability and facets of personality could have two main benefits. The first potential benefit is substantive or theoretical. Examining narrower and circumscribed facets of personality and types of cognitive ability may help to understand how the associations observed at domain level come about. Facet-level associations are likely to be more informative with regard to the specific mechanisms and processes responsible for these associations. The second potential benefit is the maximization of predictive validity. The concept of Brunswik symmetry—that is, a match between the breadth and correspondence of the predictor and the criterion constructs—as outlined by Wittmann and Süß [47] directs attention to the importance of considering the conceptual correspondence between traits and, in particular, their level of aggregation and abstraction. The strongest predictive relationships are likely to emerge between traits that are on the same level of aggregation and abstraction. As it is often hard to determine a priori what the level of abstraction of a given construct is, it can be highly instructive to compare different aggregation levels of the same construct (e.g., personality domains and facets) in terms of their predictive validity for a particular outcome.

Despite the insights that a facet-level perspective might offer, very few published studies have comprehensively investigated the associations of personality facets with different types of cognitive ability, such as fluid and crystallized intelligence. Instead, almost all existing facet-level research to date has concentrated on a subset of personality domains only—typically, a single domain. For example, Moutafi and colleagues [48] showed in their research on fluid intelligence that only the *ideas* and *actions* facets of Openness were positively correlated with this type of intelligence and that the Consciousness facets *order*, *self-discipline,* and *deliberation* were negatively correlated with it. Partial support for these findings was provided by a study by Zimprich and colleagues [49], which yielded mid-size effects of two aspects of Openness—*intellectual interests* (measured with items representing the facet *ideas*) and *unconventionality* (measured with items representing the facets *actions* and *values*)—on both fluid and crystallized intelligence. In what appears to be the only exception to this rule, Graham and Lachman [50] investigated the links between the facets of the NEO Personality Inventory (NEO-PI-R^2^; [51]) and multiple cognitive domains. However, they did not report the complete array of relationships between Big Five domains and facets and the different dimensions of cognitive ability, but rather they focused on a subset of statistically significant relationships. Moreover, their study was based on a small sample of adults (*N* = 154). This may have restricted the range of several of the measures investigated [4] and limited their statistical power.

### 1.2. The Present Study

To obtain a more nuanced and complete picture of the relations between personality and cognitive ability, the present study investigated the associations of personality domains and their facets with both fluid and crystallized intelligence. In so doing, we aimed to determine whether examining facet-level associations allows a better understanding of the cognitive ability–personality interface and provides higher predictive validity than examining only global-domain-level associations. We devoted particular attention to exploring whether—in some personality domains—the various facets showed differential associations with the two types of cognitive ability. This allowed us to gain insights into how (i.e., through the workings of which facets) the typically reported domain-level associations came about.

Based on previous findings [5,21], we expected personality traits to be more strongly related to crystallized intelligence than to fluid intelligence. Following the investment theory of intelligence [8,11], this should hold true especially for the domain of Openness, as an investment of personality in the acquisition of ability is postulated for that domain. Our research design investigating the two types of intelligence allowed us to test this assumption.

Finally, as previous studies in this area have often been criticized as being biased because they used small and often selective samples (e.g., [4,6]), we based our study on a heterogeneous population sample, thereby enhancing the generalizability of our findings and safeguarding against restrictions of range.

## 2. Method

### 2.1. Sample

Respondents of the present study participated in an online access panel conducted by Respondi AG, a German commercial provider of online panels. The present assessment was conducted in December 2016. The sample was a quota sample (based on age, gender, and education) aligned with data from the 2011 Census in Germany. Participants who either failed an attention check question or answered items in less than 3 s or in more than 30 s, on average, were excluded from the analysis sample. This resulted in an initial sample size of *N* = 365 (50% female) with an average age of 43.40 years (*SD* = 14.34 years).

### 2.2. Measures

Cognitive ability was measured using two different short tests, one assessing fluid intelligence and one measuring crystallized intelligence.

#### 2.2.1. Fluid Intelligence

As a measure for fluid intelligence, the short-scale version of the Hagen Matrices Test (Hagener Matrizen-Test, HMT-S; [52]) was used. This test comprises six matrices speeded to two minutes each. Initial validation studies confirmed high convergence of the HMT-S with the original full-scale version—which comprises 20 matrices—as well as with other tests measuring fluid intelligence (see [50]). In the present sample, the six items showed internal consistency (coefficient alpha) of 0.63.

#### 2.2.2. Crystallized Intelligence

To measure crystallized intelligence, we used the short-scale version of the Berlin Test for the Assessment of Fluid and Crystallized Intelligence (Berliner Test zur Erfassung Fluider und Kristalliner Intelligenz, BEFKI; [53]). This abbreviated version (BEFKI GC-K; [54]) assesses only crystallized intelligence. It comprises 12 knowledge items from the domains of the natural sciences, the social sciences, and the humanities. The 12 items are completed without a fixed time limit. The average completion time was approximately five minutes. In a validation study, the authors of the scale proved a clear unidimensional structure and found support for the expected correlations with educational attainment and socioeconomic status (see [54]). In the present sample, the internal consistency (coefficient alpha) of the 12 items was 0.67.

#### 2.2.3. Personality

Personality in terms of the Big Five domains and their facets was measured with the German adaptation of the 60-item Big Five Inventory–2 (BFI–2; [44,55,56]). The BFI–2 allows personality to be measured in a hierarchical way with three facets for each of the five domains. The items were formulated as short—mostly behavioral—self-descriptions (e.g., “I am compassionate, have a soft heart”; for the full list of items, see Danner and colleagues [55] for an English description of the items per facet see http://www.colby.edu/psych/personality-lab/). Items were answered on a fully labeled 5-point rating scale ranging from *fully disagree* (1) to *fully agree* (5). Internal consistency (coefficient alpha) of the domain scales ranged between 0.80 and 0.89 in the present sample. For the facets, which comprised only four items each, the alpha coefficients ranged between 0.57 and 0.84.

Descriptive statistics for all measures investigated are reported in Table 1. 

## 3. Results

**Figure 1:**
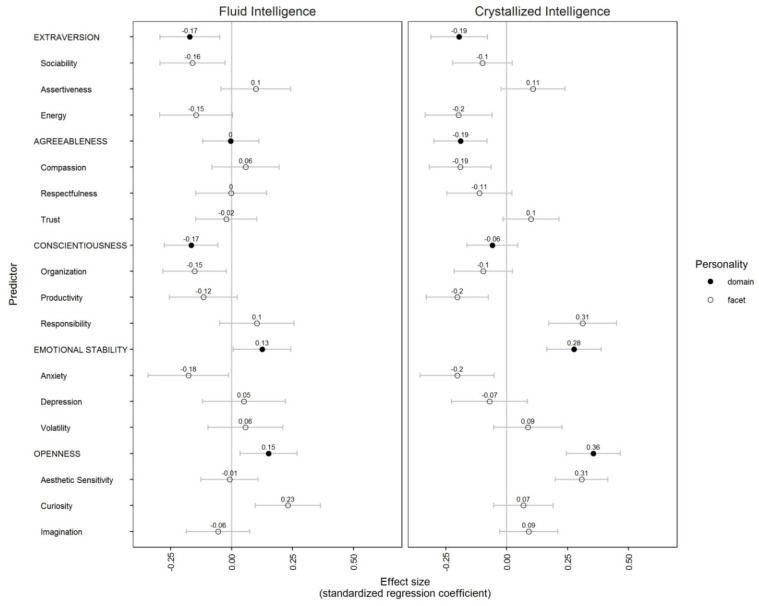
Associations between Big Five dimensions (black dots; from Model 1) and facets (from Model 2) with fluid intelligence and crystallized intelligence. Points represent standardized regression coefficients; error bars represent 95% confidence intervals.

We aimed to investigate the extent to which personality—in terms of the Big Five domains and their facets—was associated with fluid and crystallized intelligence. In particular, our aim was to examine (a) the degree to which the facets of the Big Five were incrementally related to fluid and crystallized intelligence, respectively; and (b) whether the associations with the facets allowed a better understanding of the typically found correlations with the Big Five domains. We therefore conducted separate regression analyses with fluid and crystallized intelligence as the dependent variables. In the first model, we included only the Big Five domains. In the second model, we included the three facets per Big Five domain. As previous studies of our own and of other authors had suggested some quadratic effects of personality on cognitive ability [26,27], we tested also for non-linear associations. For both models, and for both dependent variables, we found no indication of any quadratic association. Therefore, in what follows, we report linear associations only. The results of these separate regression analyses are shown in Table 2. To facilitate side-by-side comparisons of domains and facets, Figure 1 visualizes the pattern of predictive relationships and provides confidence intervals. Our results revealed, first, that the Big Five domains jointly (Model 1) were more strongly predictive of crystallized intelligence than of fluid intelligence. The amount of variance (adjusted for the number of predictors) in crystallized intelligence explained by the Big Five domains was 14%, which was more than three times higher than that for fluid intelligence (4%). This is reflected also in the magnitude of the associations of the different personality domains with the two types of intelligence. In most cases, these associations were at least twice as high for crystallized intelligence as for fluid intelligence.

Results for the associations of the various Big Five domains with cognitive ability by and large support previous findings. Openness was positively related to both types of intelligence. Across the non-speeded crystallized intelligence test and the speeded fluid intelligence test, we consistently found a negative association with Extraversion. However, the typically found negative association of Conscientiousness with cognitive ability could be confirmed only for fluid intelligence but not for crystallized intelligence. In addition, crystallized intelligence was positively associated with Emotional Stability and negatively associated with Agreeableness, whereas fluid intelligence was not.

In a second step, we investigated the differential associations of the Big Five facets with the two types of intelligence. On a general level, results first of all showed that the amount of variance in both fluid and crystallized intelligence explained by the 15 facets was twice as high as that explained by the Big Five domains. Second, results again revealed that personality explained more of the variance in crystallized intelligence than in fluid intelligence. The amount of variance in crystallized intelligence explained by the 15 facets (23%) was almost three times higher than the amount of the variance in fluid intelligence explained by these facets (8%).

Do the associations with the Big Five facets yield a more fine-grained picture and thus a better understanding of the associations of personality with cognitive ability? Our results clearly support an affirmative answer to this question. In the case of every individual Big Five domain that showed a significant association with intelligence at the domain level, this association was reflected only in a subset of the facets of that domain. For example, the positive domain-level association between Emotional Stability and crystallized intelligence was due primarily to a negative association with the facet *anxiety*, which supports the assumption that this association is primarily a function of test anxiety. For fluid intelligence, the negative association with Conscientiousness can be explained primarily by a negative effect of the *organization* facet of that domain but not by the *responsibility* facet.

Furthermore, different facets of the same domain were indicative of fluid and crystallized intelligence. Whereas, at domain level, both types of intelligence were unequivocally positively related to Openness and negatively related to Extraversion, fluid and crystallized intelligence seemed to be triggered by different facets of these domains. In the case of Openness, the facet *intellectual curiosity* was associated with fluid intelligence, whereas the two other Openness facets showed more or less zero associations. In the case of crystallized intelligence, by contrast, only the Openness facet *aesthetic sensitivity* showed a significant association. The negative association of Extraversion and fluid intelligence was due primarily to the facet *sociability*, whereas for crystallized intelligence the negative association with *energy* was about twice as large as the associations with the two other Extraversion facets.

Finally, in some cases the facets of one Big Five domain showed even inverse associations with intelligence, thus underestimating the domain-level association. Conscientiousness, for example, showed a non-significant domain-level association with crystallized intelligence. However, the facet-level analysis revealed strong associations between this type of intelligence and two facets of Conscientiousness. The Conscientiousness facet *responsibility* showed a strong positive association with crystallized intelligence, whereas the facet *productivity* was significantly negatively related to this type of intelligence.

## 4. Discussion

The present study represents one of the first comprehensive inquiries into the associations of personality facets and domains with two key types of cognitive ability, namely fluid and crystallized intelligence. Our analyses in a heterogeneous sample of the adult population in Germany clearly support the added value of investigating personality facets in addition to the typically studied personality domains and of differentiating also on the side of cognitive ability.

Three key insights emerged from our analyses. First, personality was more strongly related to crystallized than to fluid intelligence. The total variance in cognitive ability explained by personality was about three times higher for crystallized intelligence than for fluid intelligence. Likewise, the specific associations between individual personality domains and facets were almost invariably higher for crystallized intelligence than for fluid intelligence. These results are in line with some previous research (e.g., [21]) and with investment theory [8,11], which assumes that personality has a greater impact on crystallized intelligence than on fluid intelligence. As suggested by previous research [36,39], this effect held true primarily for the association with Openness.

Second, personality facets explained a larger share of the variance in both crystallized and fluid intelligence than did domains. Despite the lower reliability of the facets, the amount of variance in cognitive ability that they explained was twice as high as that explained by the domains (even after adjusting for the number of predictors in the model). These findings attest to the added value for prediction offered by a facet-level perspective.

Third, the associations of different facets from within the same domains with cognitive ability often varied widely. Some facets even had inverse relationships with cognitive ability, thereby questioning the causal unity of the corresponding domain (see [42]). From a methodological point of view, this suggests that one reason for the above-mentioned higher predictive power of personality facets compared to domains is that differential associations of different facets partly cancel each other out, thereby reducing—or even suppressing—the domain-level associations. In particular, our findings indicate that the associations with cognitive ability observed at domain level are driven only by specific facets of the respective domains. For example, we could support previous findings suggesting that *intellectual curiosity* is the Openness facet primarily positively related to fluid intelligence and *organization* is the Conscientiousness facets primarily negatively related to fluid intelligence [48,49].

As no facet-level associations have been comprehensively reported to date for the other personality domains, our results in this regard cannot be compared to an existing body of evidence. However, previous research—including measures of test anxiety—suggested that the positive association between Emotional Stability and cognitive ability was primarily a function of test anxiety [46]. Therefore, we expected to find the strongest association between cognitive ability and the *anxiety* facet of the Emotional Stability domain. This was supported for both fluid and crystallized intelligence by our data.

With regard to Conscientiousness, it has been assumed that less intelligent individuals compensate their lower abilities with high levels of Conscientiousness [9,27]. Our data revealed that this seemed to hold true primarily for the less malleable fluid intelligence factor, which—in line with previous research—can be interpreted as to be compensated primarily by high levels of *organization*. For crystallized intelligence, we found—in addition to a negative effect of *productivity* that is also interpretable as indicating compensatory behavior—a positive link with the *responsibility* facet of Conscientiousness, which may indicate that individuals who are higher in crystallized intelligence are more reliable due to their cognitive skills.

For Extraversion, it has been hypothesized that the link with cognitive ability is a function of test characteristics and that introverts perform better on tasks requiring insight and reflection [18]. Our findings provide further support for this assumption: For the non-speeded crystallized intelligence test we found that introverts clearly performed better than extraverts. This seems to have been due primarily to the higher energy level—and thus to the lower endurance—of extroverts compared to introverts.

The results reported here constitute a first systematic effort to investigate the facet-level associations of personality with cognitive ability. The scope of our findings is, however, limited in several respects. First, only short scale measures were used as indicators for fluid and crystallized intelligence. Further research should therefore aim to include more comprehensive tests. Second, the assessment was part of an online survey, and test conditions were therefore not controlled. Third, although the sample was heterogeneous with regard to key sociodemographic variables, it was limited in size. As a consequence, we were unable to utilize more sophisticated analytical tools. In particular, latent-variable analyses that account for (un-)reliability of all measures would be valuable, but were not feasible with the sample at hand. Moreover, larger sample sizes would allow for more advanced regression approaches such as cross-validation and penalized regression (for a recent application, see Seeboth and Mõttus [57]. With larger samples at hand, future studies applying these more advanced techniques could also shed light on how cognitive ability is related not only to personality domains and facets, but also to personality *nuances* (e.g., using the BFI-2 single items) as advocated by Mõttus et al. [58]. This would be conceptually beneficial, as it would allow for obtaining an even more fine-grained picture of the personality–ability relations. Fourth, it would be desirable to test the associations between personality and intelligence based on a longitudinal design, thus allowing causal effects of personality on intelligence and vice versa to be identified. Such longitudinal designs could also get us a step further toward disentangling the different directions of influence that might drive the personality–ability relations observed in cross-sectional data: effects of personality on cognitive ability; effects of cognitive ability on personality; or the common influence of third-variables (e.g., religiosity or sociodemographic status; see, e.g., [29,59].

Taken together, our initial findings on the associations of personality facets and domains with the two types of intelligence—fluid and crystallized—clearly support the added value of a more nuanced and fine-grained facet-level perspective on the personality–cognitive ability interface. The facet-level effects that we found lend further support to previous theoretical assumptions on causes of the typically observed associations between personality and cognitive ability. In addition, they also shed light on differential associations of the facets from the same domains with cognitive ability, which, in some cases, were found to reduce or even suppress the domain-level associations. Against this background, the constantly reported domain-level associations must be questioned or re-interpreted in some cases. Furthermore, the share of the variance in both crystallized and fluid intelligence explained by the facets was markedly higher than that explained by the domains. From a general point of view, our findings thus support the added value of including personality facets when investigating the associations with cognitive ability. This added value relates both to the predictive power of these facets and to the insights that they provide into the specific mechanisms and processes driving these associations.

## Figures and Tables

**Table 1:** Descriptive Statistics for the Big Five Domain and Facet Scales as well as for the Intelligence Variables.

	Cronbach’s Alpha	*M*	*SD*
Extraversion	0.86	3.19	0.64
- Sociability	0.78	3.16	0.84
- Assertiveness	0.75	3.19	0.75
- Energy	0.68	3.22	0.69
Agreeableness	0.80	3.73	0.51
- Compassion	0.71	3.95	0.67
- Respectfulness	0.65	4.06	0.59
- Trust	0.57	3.18	0.65
Conscientiousness	0.85	3.65	0.60
- Organization	0.84	3.67	0.86
- Productivity	0.69	3.51	0.68
- Responsibility	0.57	3.77	0.59
Emotional Stability	0.89	3.24	0.70
- Anxiety	0.67	3.00	0.71
- Depression	0.84	2.61	0.89
- Volatility	0.78	2.68	0.81
Open Mindedness	0.84	3.31	0.66
- Aesthetic Sensitivity	0.69	3.00	0.98
- Curiosity	0.81	3.46	0.74
- Imagination	0.81	3.46	0.79
Fluid Intelligence	0.63	2.06	1.59
Crystalized Intelligence	0.67	7.90	2.49

Note. Fluid intelligence varied between 0 and 6 correct responses, crystallized intelligence between 0 and 12 correct responses; *N* = 365.

**Table 2:** Fluid and crystallized intelligence regressed on the Big Five and their facets.

	Fluid Intelligence				Crystallized Intelligence			
Predictor	β	*SE*	*P*	*adj. R*²	β	*SE*	*p*	*adj. R*²
Model 1				0.04				0.14
Extraversion	**−0.17**	0.06	0.0066		**−0.19**	0.06	0.0011	
Agreeableness	0.00	0.06	0.9528		**−0.19**	0.06	0.0008	
Conscientiousness	**−0.17**	0.06	0.0033		−0.06	0.05	0.2728	
Emotional Stability	**0.13**	0.06	0.0383		**0.28**	0.06	<0.0001	
Openness	**0.15**	0.06	0.0116		**0.36**	0.06	<0.0001	
Model 2				0.08				0.23
Extraversion								
- Sociability	**−0.16**	0.07	0.0195		−0.10	0.06	0.1125	
- Assertiveness	0.10	0.07	0.1714		0.11	0.07	0.1058	
- Energy	−0.15	0.08	0.0582		**−0.20**	0.07	0.0052	
Agreeableness								
- Compassion	0.06	0.07	0.4163		**−0.19**	0.06	0.0035	
- Respectfulness	0.00	0.07	0.9873		−0.11	0.07	0.103	
- Trust	−0.02	0.06	0.7357		0.10	0.06	0.0893	
Conscientiousness								
- Organization	**−0.15**	0.07	0.0235		−0.10	0.06	0.1167	
- Productivity	−0.12	0.07	0.1043		**−0.20**	0.07	0.002	
- Responsibility	0.10	0.08	0.1816		**0.31**	0.07	<0.0001	
Emotional Stability								
- Anxiety	**−0.18**	0.08	0.0366		−0.20	0.08	0.0091	
- Depression	0.05	0.09	0.5626		−0.07	0.08	0.3767	
- Volatility	0.06	0.08	0.4677		0.09	0.07	0.2241	
Openness								
- Aesthetic Sensitivity	−0.01	0.06	0.8896		**0.31**	0.06	<0.0001	
- Curiosity	**0.23**	0.07	0.0008		0.07	0.06	0.2723	
- Imagination	−0.06	0.07	0.4042		0.09	0.06	0.1365	

Note. Standardized regression coefficients (β). Coefficients that were significant at least on the 0.05 level are set in bold; all tolerance values were >0.34; *N* = 365.

## Footnotes

1 In a similar fashion, the often reported negative association between religiosity Conscientiousness [28] and cognitive ability has been explained as a compensatory process. It has been assumed that people with lower cognitive abilities might be more likely to resort to the psychological and social support of religion–especially the cognitive closure that its normative orientations and explanations of existence may provide–in order to improve their well-being [29].

2 Although the authors reported that personality was measured using the NEO Five Factor Inventory (NEO-FFI), they described using the 240-item inventory, which allows facets scores to be computed on the basis of eight items per facet. This can only mean that the 240-item NEO-PI-R was used, because the NEO-FFI comprises only 60 items in total.
